# Additional Remarks on Strictures in the Urethra

**Published:** 1809-04

**Authors:** John Roberton


### *Additional Remarks on Strictures in the Urethra.*

By JOHN ROBERTON, M. D.

"It is much more congenial to every liberal mind to praise extraordinary excellence, or even to acquiesce in the narrow limits of indolent mediocrity, than to have the disagreeable necessity of expressing disapprobation, and severe censure, on dangerous prejudices and fatal presumption."

PRACTICAL considerations connected with diseases of the generative organs, have long been involved in unnecessary obscurity and even mystery; and instead of tending to develope these matters, and thereby rendering them easily understood, it rather seems to have been the wish of those engaged in such practice to make them appear complicated, obscure, and likely to be successfully treated only when under their own immediate direction, and by some peculiarity of practice with which they alone were acquainted.

I once proposed making a general review of the best of the numerous books which have, of late years, been written on stricture in the urethra. I now however find, that the opinions of every author, respecting the points which seem to me to demand greatest attention, namely, the practical ones, are so nearly alike, that a plan of this extensive kind would not only be uninteresting but unnecessary.

Instead of ascertaining the *real nature* of the disease, *a priori,* and of the plans which ought at an early period to be adopted, for the purpose of preventing the urethra from assuming that state of action which might possibly require the application of caustic for its recovery, they seem rather to have taken it for granted, that such a state existed from the first, and their whole attention has been directed to the invention of some sort of instrument, or of some new substance, for its removal.

As then the publication of Mr. Home's works on that subject has given rise to much controversy, and (as a man of honour, and one solely anxious for the progress of medicine I declare it) in my opinion, to much unnecessary torture to many individuals, I shall, in the course of this paper, occasionally take notice of those parts in which, to me, such practice appears to have been unskilfully applied. As these cases, no doubt, are the very best which the advocate for the caustic practice could adduce, and are told in his best way, as illustrating his opinions respecting these diseases, his doctrine will be seen to advantage; nor can any criticism upon them be considered as misapplied. In that gentleman's practice, many cases of a less decisive nature must have occurred; and as I can have no opportunity of examining these, I must leave to the thinking part of the community to conjecture the probable number of unsuccessful ones which must be unpublished, from those which I shall point out, now in the hands of every one. This, however ungracious the task, I am constrained to do, in order that the world may judge of the truth of the principle upon which they have been treated. Of personal motives, in the present instance, I can have none; our practice can never interfere; I am only desirous of rectifying an important part of medical practice.

It is only necessary for me to take a wrong view of the subject of which he treats, to render all his speculations and proceedings, however ingenious, respecting it, less or more erroneous. Such I do not hesitate to say has been too often the case in reasoning upon and in treating diseases in general, and I am sure in none so much as those termed strictures in the urethra, rectum, &c.

Many years ago, when reading Mr. Home's book on the subject of strictures, while I was pleased with the simplicity of his language and the plainness of his descriptions, I could not help thinking, that in the treatment of many of those cases with which he has favoured the public, in illustration of his doctrines, he had applied that harsh and I must say repulsive mode of practice, where, even from the circumstances which he himself has stated, he had no right to adopt; nay, farther, were I disposed to doubt Mr. Home's authority, I should be inclined to pronounce, that, under certain circumstances connected with some of his cases, no such result could be obtained from the application of caustic bougies. In short, I conceive it a very exceptionable work.

These opinions respecting the application of caustic to the urethra, remained with me for some time little more than conjecture, as I then had no opportunity of ascertaining the certainty of them from practice. Since that time, however, I have not only had ample opportunity of confirming them by experience, unbiassed by any particular hypothesis or theory, but facts have crowded upon me from every quarter, to convince me that *permanent* stricture in the urethra is a very uncommon disease, and that Mr. Home's Treatises upon that subject are even more faulty faulty than at first reading I conceived them to be; yet, strange to tell, a man can scarcely be recognized, at least in the fashionable world, as properly initiated, unless he has submitted to burning by caustic.

Since my publication on the Internal Use of Cantharides appeared, I have had numerous opportunities not only of confirming the doctrines there advanced, but of extending my views on that subject to a greater extent than I at that time ever expected. My experience on that subject is not only general, but, on the principles I then hastily delivered, uniformly successful; and the numerous favorable communications which I am daily in the habit of receiving, convince me, that in a much shorter time than important improvements in the practical part of our art commonly meet with public sanction, this practice will, under proper regulations, become still more general, and more successful.

It must appear to every person, that when one particular mode of treatment is uniformly adopted for the removal of disease, and pertinaciously persisted in, in spite of observation and of reason, we may rest assured that the department of science, which ought to regulate that particular part of our art, is in a very imperfect state.

It has always seemed to me extremely proper, when a man submitted his opinions to the world, that those opinions should be examined, and only held valid where unbiassed reasoning could support them. I can have no wish, I may again observe, to object to Mr. Home's doctrines, as a private individual; indeed, in that capacity, I should never have thought of making a single proposition respecting them: but as a servant of the public, as one engaged in the arduous task of relieving human misery in the easiest way for the sufferer, I deem myself perfectly at liberty, even without attempting to offer an apology, to make such remarks on the opinions and practice as I may think proper. I invite the public to do the same by myself; provided they are reasonable and deserving of notice, I shall listen to the remarks and criticisms with pleasure; and if it be proved that the opinions I have submitted to the public, on various occasions, as well as those I may yet submit, are faulty or imperfect, I shall not be offended, but on the contrary, pleased either to admit, in the same public manner, of such observations, or to correct them with all imaginable dispatch.

Stricture in the urethra seems to me a disease much easier understood as well as removed, than one would expect pect from the immense multitude of pamphlets, papers, and volumes which have been written upon it, as well as from the controversies and disputes which the very name of this disease has given origin to. In short, the many publications which this disease has occasioned, clearly demonstrates that much misunderstanding exists respecting its real nature, even among those who pretend to teach others every thing relating to it.

As stricture in the urethra of any kind and healthy action in the same part, are incompatible with each other, the first object of the surgeon is, during the existence of stricture, to ascertain, which he may do by unbiased examination in the greater proportion of cases, what the particular action is which preponderates in the urethra at the time. Spasmodic action may exist in a violent degree, independently of any cause which we can assign for its presence; it may also exist in consequence of long continued debility of these parts, and be preceded, accompanied, or even followed, either by gleet, seminal emissions, or total impotency. Permanent stricture may also exist in consequence of long continued spasmodic stricture occasioned by any of the preceding causes; but it is most generally induced, in a greater or less degree, by the rude application either of common or caustic bougies.

### Of Spasmodic Stricture.

Spasmodic stricture is an affection of a very different nature from permanent stricture; its approach is in general sudden, and often very violent; it may appear in various parts of the urethra at the same or at different periods; and seldom if ever, till length of time or the improper application of irritating substances to them, do they become of a permanent nature. They never, if properly treated, require the application of caustic substances for their removal, but may, in every instance, however severe, be removed by other means.

The existence of spasmodic stricture is not alone indicated by its suddenly and almost entirely obstructing the urethra, and by its departure taking place in the same sudden way; spasmodic contraction may be various in its severity, as well as in its extent and duration; and, if this is well understood, it may, in most cases, account for the irregularity of time which is often observed in its approaches, as well as in its disappearance. The greater proportion of the cases, I may observe, related by Mr. Whately,

Whately, in his pamphlet on Strictures, were purely spasmodic. In some of them, however, permanent strictures, and in others an irregularity of the canal of the urethra, were formed solely by the use of the caustic bougie. Mr Wadd's cases were evidently all of a spasmodic nature; indeed, he wishes to prove this; but in many of them, the spasm did not exist previous to the introduction of his bougies. And that Mr. Home's were generally so also, I shall endeavour to show. An unprejudiced perusal of these cases will at once convince any one of the truth I now state.

Previous to my investigations respecting the cure of gleets, they were deemed irremediable, unless they yielded to the most common, though at that period the only, means of cure which were known. When, under these circumstances, as almost always happened, the gleet could not be removed, a bougie was instantly introduced to ascertain if it existed in consequence of stricture; and the spasmodic contractions caused by this application gave rise to a belief, that these contractions were permanent strictures; and this again occasioned a train of cruel practice.

Spasm, however, is apt to occur in every part of the urethra from its external orifice to the bladder; nor does it seem much influenced by the action of the muscles surrounding a part of the canal. It has been supposed to exist most frequently in those parts nearest the bladder, but my experience does not warrant me to draw such a conclusion; for I have found it equally severe in every part of that canal. What must have greatly contributed to the opinion that spasm was most frequent nearest the bladder, is the length and curve of the urethra, which even in its healthy state may present various obstructions to the passage of the bougie; or they may have been caused there by the irritating property of that instrument.

It is remarkable Mr. Home, in mentioning the usual situation of strictures in the urethra, (see page 28), assures us, that in his practice there was always one stricture about seven inches from the external orifice, whether there were others or not. In other words, I should say, the most common situation for *permanent* stricture is certainly to be found at that part of the canal where, from its curvature, there is the greatest difficulty in passing the bougie.

It is curious also, that in parts similarly constructed, in some respects, to the urethra, strictures have not been more more frequently discovered; and it is not improbable, that this may be owing to the parts, such as the whole intestinal canal, &c. not being so liable to surgical treatment. It would bring no merit to the present numerous operators for the removal of *permanent stricture*, even to hint at the possibility of that disease existing in parts, such as those mentioned above, where surgeons could not apply *their* means of cure. A bold step, however, has been made toward this in the attempts which have been practiced to persuade people they were affected with stricture in the ends of this canal, (because to them the caustic could be applied,) viz. the rectum and œsophagus; when it is probable some hæmorrhoidal tumours were the cause of the one, and globus hystericus the cause of the other.

Mr. Benj. Bell, whose experience was certainly great, in speaking of the effects of gonorrhœa, observes, that " the irritation produced in the urethra by it, is, in some cases, so very great as to excite contraction of the passage in a very distressful degree. I have known," says he, " the urine so completely obstructed by this alone, as to give cause to suspect that strictures were formed of the most alarming nature; in which, neither staff, catheter, nor bougie, could be introduced, but with more force than can ever with safety be applied." Mr. Home, in his opinion respecting the existence of permanent stricture and his practice for its removal, is never so particular. I may remark that it is alone by the discernment of the medical attendant, that these different states of disease are to be known, and consequently their entire removal scientifically effected.

When the parts, by their diseased action, or by some peculiarity of constitution of the individual, had assumed a violently spasmodic action, and when the want of patience or want of discernment of the surgeon, mistaking in practice all such obstructions for permanent stricture, and acting accordingly, often reduced the unfortunate patient to a state of great wretchedness and misery.

Certainly, when we reflect on the many causes to which the strictures of various kind and degree owe their origin, and by which they have their existence prolonged, we must at once perceive the absolute necessity there is for considering and relieving them in some scientific way; not by the Farrier-like, and I would say, inhuman methods too commonly practiced at the present moment. Under these dreadful plans of practice, it is inconsistent with every reasoning faculty of the human mind to suppose pose that they should be so often successful as they are represented to be, and that none of those masses of human misery, certainly occasioned by it, but always artfully attributed to other causes, should owe their entire existence to these plans alone; and this I shall endeavour to show.

### Cure of Sapsmodic Stricture.

It is a curious fact, which I cannot avoid introducing, that, since my practice in diseases of the generative organs has been rather upon an extensive scale, almost every gentleman who consulted me, did so in consequence, as he asserted, of being affected with permanent stricture. I, however, found that that disease was scarcely ever the one with which he was affected; that the various sensations he complained of, arose principally from debility of these parts or from strictures, purely spasmodic, and removeable only by the use of stimulating medicines, or by antispasmodic medicines internally administered or externally applied.

Mr. J. Hunter observes, that "when a bougie can readily pass, there is no necessity for using any other method to remove the stricture;" and I certainly would add to this, that when the urine can flow in a full stream through the canal, there is no necessity for applying either the one or the other, although in Mr. Home's book we find many cases in which he applied caustic when the stream was sufficiently large. A bougie, however, in some of these cases being introduced, brought on spasm, for the removal of which he applied caustic, and, as he observes, the patient, in some cases completely, in others nearly recovered.

It is very evident from the language even of the advocates both for the application of caustic, and for the use of the common bougie in the removal of stricture, that the contraction of the parts is very apt to return, and it is allowed, that a relapse after burning with caustic is uniformly worse than after the use of the simple bougie. Thus it is evident from those on both sides of the question, that their applications are more calculated to remove the effect of the contracting power, than the cause of that disease. Indeed, no topical application can *alone* effect this purpose. The writers on these subjects want something, but they do not know what they want; some indeed, recommend internal medicines and external applications, for the relaxation of strictures; but these substances are recommended in so ill-arranged or contracted tracted a plan, that they cannot be used with decided advantage.

Although from various circumstances we may be able, with tolerable certainty, to ascertain whether an obstruction in the urethra has arisen from permanent or from spasmodic stricture, yet the chances of deception, from the similarity of these diseases in many instances, ought to induce us, *in every case*, to try every means for the removal of the spasm, lest that should be the sole disease, previous to our use of the caustic. This, I say, ought *at all times* to be done, notwithstanding Mr. Home's arguments, that burning these parts is attended with *little or no pain, and far less risk.*

Instead of waiting for the relief of the spasm by internal or external applications, particularly if the case be urgent, Mr. Hunter recommends, in page 164 of his book on the Venereal Disease, the immediate application of the catheter or bougie. Even under these circumstances, I think this practice extremely harsh, as it must at all times not only occasion the most excruciating pain and a greater degree of contraction, but, in perhaps every instance, must, in a greater or less degree, rupture the parts, and thus rather accumulate than remove the evil. The steady use of internal medicines and external applications are therefore indispensibly necessary; and if the urgency of the symptoms will not allow us to wait their effects, it were better that we should puncture in the perinæum or rectum beyond the contracted portion of the urethra, than follow the plan recommended by Mr. Hunter.

Mr. Hunter, however, by no means wanted a knowledge of the power of antispasmodic medicines; but, with the order of their application for the removal of stricture, he does not seem to have been acquainted; for, under the very circumstances, where their use was absolutely necessary, he does not once hint at the propriety of their employment. "The time," says he, in page 121, "that each bougie should remain in the passage must be determined by the feelings of the patient, for it should never give pain if possible. Going beyond this point is destroying the intention, increasing the very symptoms that are meant to be relieved, and producing irritation, which for a time, renders the further application of the bougie improper." Now, had Mr. Hunter made the proper applications previous to and during the employment of these bougies, many, perhaps all, of these distressing symptoms, would never have been experienced, and the bougie gie consequently might have been continued with impunity, with perhaps few, if any interruptions to its use, till the cure rendered its further application unnecessary. Neglect of these considerations did not alone exist with Mr. Hunter; for every one who has written on the dilatation of stricture by the simple bougie has similarly erred. Mr. Hunter, in the same page, evidently only wanted these means to insure his complete success in the cure of such strictures. Even by his cautious management, he succeeded in the removal of many without them. He here observes, that "the bougie should be increased in size according to the facility with which the stricture dilates, and the ease with which the patient bears the dilatation. If the parts are very firm, or very irritable, the increase of the size of the bougie should be slow, gradually stealing upon the parts, and allowing them to adapt their structure to the increased size." Although, however, those strictures were dilatable without any means being applied to prepare the system for such practice, it does not follow that such means are unnecessary, but highly proper, as affording an easier method of cure than the one formerly in use.

Mr. Whately justly observes, in page 63 of his pamphlet respecting Mr. Home's practice, that "it is essentially necessary, in our present imperfect acquaintance with the caustic, to endeavour to dilate all strictures of the urethra by means of common bougies, before any attempt be made with the caustic to effect their cure." This is a most important point, and I am sorry to say, that *in practice* it is seldom, indeed scarcely ever, attended to, and when such a method of cure has been adopted, even under the circumstances Mr. Whately alludes to, from the system not being properly prepared by internal medicines and external applications, it is scarcely ever attended with *permanent* benefit; indeed, on the contrary, by thrusting a bougie into the urethra, with sufficient force to distend the contracted part, we often increase the distress, but seldom if ever relieve it.

I wish particularly to insist on this, that either pressure by a bougie, or the *wedging* process, which are the methods recommended for their dilatation, are, in a great majority of cases, impracticable, unless proper measures have been previously adopted to suit the system for such practice. I allow that in *some cases*, simple pressure will effect a cure, but it must always occasion much more pain and be more slowly, and after all less perfectly removed, than when these means are assisted by internal applications, &c.

The

The attempt indeed to introduce a bougie for the dilatation of a spasmodic stricture, particularly if violent, before the application of external and internal medicines have been made to prepare the parts for such an application, must be attended with most excruciating pain, and will seldom, indeed, I believe never, remove the affection. By a bougie being introduced in the most trifling cases of spasmodic stricture, before other means have been judiciously employed, the disease has often been rendered actually much worse, and this at length has given rise to the existence of permanent stricture in these parts, and the application of the caustic has then been necessarily adopted in numerous instances for their removal.

It may then be considered as a general rule, that the application even of the common bougie, without previously adopting those plans which must prepare the system in general, and the urethra as a part of the whole, for its application, must always increase the irritable state of that canal, and consequently render the disease worse. This is a point to which we cannot pay too much attention.

As one proof that it is not the number of medicines, but the order and method in which they are applied, that is most beneficial in the removal of spasmodic stricture, I may mention, that previous to my having arranged them, as I now do, I was in the habit of applying all the different articles recommended by authors, in the common confused and indiscriminate way, but never, unless in slight cases, effected any permanently good purpose. My experience, however, warrants me to vouch for the effects of these medicines, internally and externally applied, in the removal of some cases which I had formerly failed in curing.

It is not then from a vast variety of external applications or internal exhibition of medicines, without any order observed in their application, which is too often the case in books written upon this subject, that we are to expect lasting or even temporary benefit. I know of no other complaint, where greater discrimination is necessary in the administration of remedies, a single error indeed may, even in the exhibition of the most useful medicines, thwart all the advantages which would have arisen from the properly regulated employment of the same medicines.

For the removal of spasmodic strictures in the urethra, we must first, provided the patient be plethoric, employ general blood-letting, and apply leeches to the part affected. At the same time we ought to employ internally as as much camphor, opium and æther, as the stomach can receive without producing sickness. After this, in from one to three or four days, according to the particular constitution of the patient, we must apply a blister under the penis, or in the perinæum, according to the situation of the stricture; and it may sometimes be necessary to apply one of a pretty large size across the loins. After one or more of these have produced their escarotic effects, we may administer an injection, *per anum,* of tobacco-smoke, or of laudanum and æther, and then lay his whole body, for about ten minutes, in a warm bath. After this, and while he still remains in the warm bath, we may inject a mixture of laudanum, oil, and æther into the urethra, and after dipping a bougie, or rather an elastic gum catheter, if it be necessary to use it, while the patient remains in the warm water, into the same mixture, we, I think, can scarcely fail of passing any spasmodic stricture that may have formerly existed in that passage. I by no means propose that all these applications shall be used at once, unless where a failure of one or more of them has previously happened. In many cases I have used one, in others two or more, and in many I have been obliged to employ them all, and even repeat them, before I effected my purpose.

The preferable position for the patient's body to be placed in, for the introduction of the bougie, is on his back, with his knees somewhat bent. In standing during this operation, which is sometimes done, the parts are apt to be compressed by the action of the surrounding muscles; and in sitting, the urethra is very apt to be so completely compressed as to prevent the possibility of introducing that instrument.

I would pointedly object to the use of Mr. C. Bell's wire bougies with a silver knob, of various size, used to ascertain the distance of stricture from the orifice of the urethra. That canal is so liable to spasmodic action, that however easily one of these bougies may be introduced to the distance the surgeon would wish, it must often be so firmly grasped by the spasmodic action, occasioned by their introduction, as to prevent its being withdrawn without too much force being applied, or at least for a considerable length of time, till the spasmodic action has given way, either of itself, or by the application of remedies for that purpose. This fact is so evident, that I am sure it only requires to be stated that it may be at once perceived.

I particularly wish it to be understood that I conceive, and from practice I have proved it, that when every means employed to relax a stricture has in part failed of what we expected, the gentle pressure of a bougie upon its anterior part, the other means still being employed, will at length admit of one being pushed through it. This practice, of *gently* pressing on its anterior part, I prefer to even the smallest bougie being *wedged* in the strictured portion itself; the first often assists in relaxing the part, while the latter, unless proper attention be paid to internal medicines and external applications, *always* makes the contraction worse.

We are certainly much indebted to Mr. Home for having introduced more generally into practice the use of the large in preference to the small bougie. Mr. Sharpe, however, in page 183, of his Critical Enquiry, was rather before him in his opinion respecting the comparative advantages of a large to those of a small instrument for that purpose. He says, "A large catheter or sound may sometimes be passed into the bladder when a small one cannot." The urethra being fully distended by the large instrument, allows it to pass easily, but with the small one its progress is obstructed by the sides of the urethra entangling its point, and in almost every instance preventing its free passage into the bladder. A small bougie then ought never to be used, except where the passage, from disease, cannot admit of a large one. In the first place, a small one scarcely ever passes so freely along the canal as one of a larger size, and, in the next place, a small one can never remain long enough in the urethra to effect any beneficial purpose till it becomes quite soft and useless.

I am further decidedly of opinion, that no attempt ought ever to be made to introduce a straight bougie into the bladder, as, from the curve of the urethra, it is at all times liable to be obstructed in its passage, and, in irritable habits, invariably it throws the parts into a state of temporary contraction. The use of these, therefore, I have entirely laid aside, and I would warmly solicit others to do so likewise. In preference to these straight bougies, I gradually soften them by applying a very gentle heat, and then bend them into the form of a common catheter, which, when allowed to cool, I have always found to answer the purposes much better than any other form.

Although in the generality of cases of spasmodic stricture, when the system has been properly managed, there is no very great resistance to the introduction of a bougie, yet there are some of them, even with attention to the above circumstances, to which considerable pressure must be made before before we can pass it into the bladder. If, however, much pain be occasioned during these trials, we ought not to persist in them, as, from that circumstance, the necessity of further relaxing the parts by internal medicines or external applications is absolutely necessary.

The bougie is often stopt by the lacunæ of the urethra, which, by those in the habit of expecting *permanent stricture*, is often mistaken for one; and thus caustic is applied to burn a passage for the bougie, when neither spasmodic action of the parts, nor the smallest diminution of the canal of the urethra, is present; at least till either one or other of these effects has been caused by the above treatment.

The preventing a bougie from rupturing the canal of the urethra, and penetrating some of the neighbouring parts while passing into the bladder, requires both dexterity and considerable experience. One, however, would be apt to believe, were they not to think for themselves, that, from Mr. Home's advice, the chances of getting out of the right road were not only very uncommon, but, except in the hands of the most stupid of the human race, almost impossible. This mode of attempting to render easy one of not the least important operations in surgery, has, I am sure, made many a fool-hardy practitioner of that art commit numerous, and in some instances almost irreparable blunders. Because Mr. Home, or any person in the daily habit of using the bougie, could do this operation with ease, and describe and recommend it to others with the same ease, they conceive it a shameful business to fail, and are consequently induced to thrust, and poke, and squeeze their instrument into any part but the right one; and even when they fail, so convinced are they still of its being as easy an operation as that of thrusting one's hand into a glove, that they have only to take the trouble to turn it off their shoulders, and conclude that there was some mal-formation of the parts, from which originated all the difficulty.

The most respectable authors, who have written on strictures in the urethra, seem fully aware of the inconvenience, and even danger attending a perseverance in the use of the common bougie, when such an application occasions great pain. Some of them rest contented in expectation of a repetition of short trials, at length rendering the parts capable of being distended in this way; while others, who however certainly do approach nearer the right method, prescribe a farrago of internal and external applications, without that simplicity, order, or regularity

 for for their administration, which alone can be attended with decided success.

Mr. Hunter, page 122, was perfectly aware of the mischief arising from wedging, or thrusting a bougie too violently into the urethra, in order to remove obstructions by the process of ulceration. "I believe," says he, "there are few patients that will submit to this practice, and indeed few will be able to bear it; for I have seen it bring on violent spasms in the part, which produced suppression of urine, and proved very troublesome." No doubt of it; and it would be well that an observance of various circumstances to prevent such practice were more closely attended to; then the excruciating pains which a patient is doomed to bear, the unnecessary application of caustic, and the irrecoverably distorted urethra, which is often the consequence of it, would all be avoided.

If, however, we should find it necessary in some cases to allow the bougie to remain in the urethra several hours, it will be absolutely necessary to fix it in some way, either to prevent its being thrust entirely out of the urethra, or, as sometimes happens, to prevent its slipping into the bladder. For this purpose, ligatures put round the bougie, and fixed to the penis or the scrotum, &c. are recommended; but I have found the least troublesome and most effectual way to be, to fix the end of the bougie into a piece of cork, or by repeatedly dipping the end of it, before subjecting it to use, into melted sealing-wax, till it has acquired sufficient size to prevent the possibility of its slipping into the bladder. To this a piece of thread, or a bag like the finger of a glove, may be applied over the penis to prevent the bougie from slipping out.

A continuance, however, of bougies for any great length of time after the canal of the urethra has been distended to its natural width, seems to me at lesst questionable, if not unnecessary. For if the disposition to contraction exists, in any violent degree, after this period, we may be assured that local applications of any kind will not remove it; that the disease of the parts can only be permanently removed by the diseased action, which, as it very commonly arises from debility, must be treated by medicines which counteract it. I have seen such a state frequently attended with considerable pain, giving rise in the mind of some more versant in name, than in the actual practice of such complaints, to a supposition of its existing in consequence of inflammation. In such cases, however, I have employed employed cantharides internally with the greatest benefit.

In some cases, I may remark, of retention of urine, from whatever cause, there often originates in the urethra the most violent spasm, to remove which the evacuation of the urine was alone necessary. For this purpose, surgeons have been in the habit of puncturing the bladder in different places, sometimes above the pubis, which is the most exceptionable, sometimes in the perinæum, and at other times by the rectum. Either of these operations usually relaxed the spasmodic action of the urethra, and the urine flowed in the natural way; but certainly a fair trial of internal medicines and external applications, so long as we could with safety delay puncturing the bladder, with properly regulated attempts to introduce a catheter or bougie, has not been made, and where such means were successful, the operation would neither be so hazardous nor so formidable.

